# Long-term complete remission following treatment with polatuzumab vedotin, rituximab, and bendamustine in a patient with mantle cell lymphoma relapsing after CAR-T cell therapy: a case report

**DOI:** 10.1177/17588359261472319

**Published:** 2026-08-05

**Authors:** Vincent Sunder-Plassmann, Barbara Kiesewetter, Markus Raderer

**Affiliations:** Division of Oncology, Department of Medicine I, Medical University of Vienna, Vienna, Austria; Christian Doppler Laboratory for Personalized Immunotherapy, Department of Medicine I, Medical University of Vienna, Vienna, Austria; Division of Oncology, Department of Medicine I, Medical University of Vienna, Vienna, Austria; Division of Oncology, Department of Medicine I, Medical University of Vienna, Waehringer Guertel 18-20, Vienna 1090, Austria

**Keywords:** case report mantle cell lymphoma, polatuzumab vedotin, salvage treatment

## Abstract

Mantle cell lymphoma (MCL) remains an incurable B-cell malignancy despite major advances in its therapeutic management. While Bruton tyrosine kinase inhibitors (BTKi) and, more recently, CD19-directed chimeric antigen receptor T-cell (CAR-T) therapy have significantly improved outcomes in relapsed or refractory MCL, relapse after CAR-T therapy is associated with a dismal prognosis and represents a major therapeutic challenge. Here, we report the case of a 43-year-old male diagnosed with stage IV common-type MCL, who achieved long-term complete remission (CR) after treatment with polatuzumab vedotin, rituximab, and bendamustine (Pola-R-Benda) following relapse after CAR-T therapy. The patient had previously received standard first-line immunochemotherapy (R-CHOP alternating with R-DHAP) and autologous stem-cell transplantation, resulting in durable CR, followed by ibrutinib at first relapse in 2017 with sustained remission until 2021. Eleven months after CAR-T therapy with brexucabtagene autoleucel, the patient experienced systemic relapse. Enrollment into a Pola-R-Benda clinical protocol resulted in complete metabolic remission after three cycles, confirmed by positron emission tomography/computer tomography. Treatment was well tolerated except for mild grade 2 diarrhea. The patient remains in ongoing CR >20 months after initiation of Pola-R-Benda. To our knowledge, this is among the first reports of long-term remission with Pola-R-Benda following CAR-T failure in MCL, suggesting that antibody-drug conjugate-based therapy may represent an effective salvage option in this highly refractory clinical setting.

## Introduction

Mantle cell lymphoma (MCL) is among the more common types of B-cell lymphoma and accounts for roughly 8% of newly diagnosed non-Hodgkin lymphomas.^1–3^ It is usually diagnosed at an advanced stage, with the majority of patients presenting with bone marrow- (70%) and GI-tract/extranodal involvement (86%) in addition to usually generalized lymphadenopathy. The genetic hallmark of MCL is the t(11;14) translocation resulting in overexpression of Cyclin D1, but genetic instability appears to occur over the course of the disease following different treatment lines, with responses becoming fewer and shorter.^4–7^

For the time being, MCL is thought to be incurable, though the clinical course may vary from highly indolent for especially leukemic nonnodal MCL to highly aggressive in pleomorphic and blastoid MCL.^8^ The mainstay of treatment in younger and fitter patients has been induction with immunochemotherapy according to the Nordic protocol (R-CHOP alternating with R-DHAP), followed by autologous bone marrow transplant and maintenance with the anti-CD20 antibody Rituximab (R). With this regimen, a median overall survival (OS) of 83.2% at 7 years has been reported.^9^ Patients, however, will inevitably relapse, and Bruton tyrosine kinase inhibitors (BTKi) as exemplified by ibrutinib have become the standard of care in second treatment in these patients, resulting in progression-free survival (PFS) rates in the range of 24 months.^7,10^ While results in patients relapsing from BTKi therapies have been poor with conventional therapies showing a median OS of 9.7 months according to the SCHOLAR-2 analysis,^11^ the advent of treatment with chimeric antigen receptor T-cells (CAR-T) has dramatically improved the outcome of such patients. The pivotal results of the ZUMA-2 study have demonstrated a median PFS of 48 months in patients achieving a complete remission (CR) following application of brexucabtagene autoleucel (Brexu-Cel).^12^ Real-world data have consecutively confirmed these results also in high-risk patients including patients with CNS involvement, TP53 aberrations, and high Ki67 values.^13,14^

Patients relapsing after CAR-T cell therapy nevertheless face a dismal prognosis, and the median PFS and the OS have been reported at 2.5 and 5.4 months, respectively, in a recent analysis.^15^ In view of this, effective treatment regimens are an unmet medical need in this situation. We report the case of a heavily pretreated patient with MCL relapsing 11 months after therapy with Brexu-Cel, who achieved long-term CR following therapy with polatuzumab vedotin, rituximab, and bendamustine (Pola-R-Benda). The reporting of this study conforms to the CARE guideline (Supplemental Figure 1).^16^

## Case report

A 43-year-old male was diagnosed with MCL (common type), stage IV due to generalized lymphadenopathy, and bone-marrow involvement in 2013. According to age and stage, the patient was treated with six cycles of immunochemotherapy consisting of alternating R-CHOP and R-DHAP followed by autologous bone marrow transplant. Treatment resulted in CR, and the clinical course was uneventful until relapse of the lymphoma in 2017. Consecutively, treatment with ibrutinib was initiated, which resulted in CR after 9 months of treatment. Ibrutinib monotherapy was continued until 04/2021, where it was discontinued for compliance reasons in ongoing CR.

In February 2022, however, computer tomography (CT) scans were again suggestive of recurrence with multiple enlarged lymph nodes in the axillary, inguinal, and retroperitoneal region. Biopsy revealed recurrence of the common type MCL, with a Ki67 of 30%. Bone marrow biopsy showed no definitive signs of infiltration with MCL.

In view of this, the patient underwent treatment with Brexu-Cel following lymphodepletion with fludarabine and cyclophosphamide. Application of CAR-T cells was tolerated well. Due to cytokine-release syndrome, two doses of tocilizumab along with dexamethasone were necessary for control, but no neurotoxicity occurred.

CT scans and a positron emission tomography CT (PET/CT) performed on day 100 after application of Brexu-Cel showed CR of the MCL, that persisted in subsequent control after 3 more months. However, a CT scan performed in January 2023 showed multiple pulmonary infiltrates, which were subjected to transbronchial biopsy and showed infiltration with a CD20 positive B-Cell lymphoma. The patient was admitted at our institution in February 2023 for further therapy. Upon physical examination, enlarged lymph nodes were found in the axilla and in the inguinal region, and excision of an inguinal lymph node was performed. Immunohistochemistry revealed a phenotype consistent with MCL, showing positivity for CD5, CD19, CD20, CD79a, SOX11, Bcl2, and Cyclin D1, with partial weak expression of Bcl6 and negativity for CD3, CD10, CD23, and CD200 at a Ki-67 of 30%. Light chain staining demonstrated weak cytoplasmic kappa restriction. These findings confirmed relapse of classical MCL. Notably, CD19 expression was retained at relapse, arguing against CD19 antigen loss as a mechanism of CAR-T failure in this patient. Although CD79b expression was not specifically assessed, CD79a expression supports preservation of B-cell receptor components and provides biological plausibility for targeting CD79b with polatuzumab vedotin.

The patient was enrolled in an academic, investigator-initiated study on Pola-R-Benda. The study was subsequently terminated early after this patient due to slow patient accrual, likely reflecting the rapidly evolving therapeutic landscape and increasing availability of CAR-T cell therapies in this setting. Treatment consisted of rituximab at a dose of 375 mg/m^2^ on day 1, bendamustine 90 mg/m^2^ days 2 and 3 of each cycle and polatuzumab-vedotin at a dose of 1.8 mg/kg on day 2 of cycle one, and on day 1 of each consecutive cycle. Cycles were repeated every 21 days. Restaging after 3 courses showed CR of MCL, and therapy was thus continued, with 6 cycles in total being planned. Apart from transient episodes of grade 2 diarrhea managed with loperamide, no side effects related to the investigational medicinal products were noted during treatment. Particularly, no cytopenias, infections, or worsening of preexisting polyneuropathy occurred. However, 10 days after cycle 5, the patient was admitted with worsening retrosternal chest pain of approximately 1 week’s duration. Electrocardiography demonstrated ST-segment depressions in leads V3–V6 with ST-elevation in aVR. High-sensitivity troponin T was elevated at 121 ng/L. Coronary angiography revealed three-vessel coronary artery disease (CAD) with a high-grade, heavily calcified long-segment stenosis of the proximal to mid left anterior descending artery (LAD) with a calcified nodule. An intravascular ultrasound-guided percutaneous coronary intervention (PCI) was performed, including rotational atherectomy followed by implementation of two drug-eluding stents in the LAD. Additional distal circumflex and small-caliber right coronary artery stenoses were not intervened. Transthoracic echocardiography after PCI demonstrated a mildly reduced left ventricular systolic function with an ejection fraction of 45% and regional wall motion abnormalities in the apical segments. The patient was initiated on dual antiplatelet therapy, lipid-lowering therapy, and guideline-directed heart failure medication. Baseline medication before the cardiovascular event consisted of low molecular weight heparin due to immobility after ankle surgery, gabapentin, and vitamin B due to persisting polyneuropathy after prior chemotherapy, as well as pro re nata paracetamol and metamizole. Control coronary angiography 15 months after the index event demonstrated no relevant re-stenosis of the intervened LAD and largely unchanged stenoses in the distal circumflex and right coronary artery. Regarding cardiovascular risk factors, the patient had a significant smoking history of approximately 30 pack-years. Prior to Pola-R-Benda initiation, no symptomatic cardiovascular disease has been documented. The temporal association with chemoimmunotherapy was carefully evaluated; however, given the presence of advanced multivessel atherosclerotic disease with severe calcification, the event was considered most consistent with progression of underlying CAD rather than a direct treatment-related effect. However, treatment with Pola-R-Benda was discontinued after the previously received five cycles. Follow-up CTs were performed every 3 months, and no signs of MCL recurrence were noted as of 11/2025. The patient remained in CR >20 months after start of treatment, with an uneventful clinical course apart from one episode of pneumonia treated with i.v. antibiotics 3 months after Pola-R-Benda discontinuation.

## Discussion

Prior to the introduction BTKis and CD19-directed CAR-T therapies, conventional salvage regimens for relapsed or refractory MCL resulted in poor outcomes, with median survival after relapse of only a few months.^11,17,18^ The therapeutic landscape has changed substantially with the availability of BTKi, such as ibrutinib, which yielded PFS durations of approximately 24 months in the relapsed/refractory setting.^7,10^ However, once patients progress after BTKi therapy, the prognosis again becomes dismal. In the SCHOLAR-2 analysis, median OS after BTKi failure was only 9.7 months.^11^ The advent of CAR-T cell therapy had been transformative in this context. In the pivotal ZUMA-2 study, Brexu-Cel achieved an ORR of 91% with a CR rate of 68%, and long-term follow-up demonstrated a median OS of 46.6 months overall, and a median PFS of 48 months in patients achieving a CR.^12^

These results illustrate the substantial therapeutic advance represented by CAR-T therapy; nonetheless, most patients ultimately relapse and face a therapeutic void, as data on optimal salvage strategies after CAR-T failure remain sparse. Real-world outcome data highlight the magnitude of this problem. In a recent registry analysis of patients relapsing after CAR-T therapy, median post-failure OS was only 5.8 months (95% confidence interval (CI): 3.2–11.3).^19^ This underscores the high-risk nature of this population and the urgent need for effective salvage approaches beyond BTKis and cellular therapies.

Against this background, the present case is notable. Our patient, a heavily pretreated 43-year-old male with MCL relapsed 11 months after Brexu-Cel. Subsequently, he achieved a durable CR lasting >20 months following treatment with Pola-R-Benda, which is remarkable given prior CAR-T failure and the dismal outlook in this setting and suggests that this regimen may provide meaningful antitumor activity even in highly refractory disease. To our knowledge, long-term CR after Pola-R-Benda in this setting has rarely been reported thus far.

The rationale for using Pola-R-Benda in this context derives primarily from experience in diffuse large-B-cell lymphoma (DLBCL). Polatuzumab vedotin is an antibody-drug conjugate (ADC) directed against CD79b, a component of the B-cell receptor expressed broadly across B-cell malignancies, including MCL. In the GO29365 trial in relapsed/refractory DLBCL, the addition of polatuzumab vedotin to bendamustine and rituximab significantly improved outcomes compared with BR alone, with a median PFS of 9.2 versus 3.7 months (HR: 0.39; 95% CI: 0.2–0.7), and a median OS of 12.4 versus 4.5 months (HR: 0.4; 95% CI: 0.2–0.7) with over 4 years of follow-up.^20^ Interestingly, the addition of polatuzumab vedotin to bendamustine and rituximab did not result in PFS or OS benefit compared to R-Benda in the follicular lymphoma (FL) arm of the GO29365 trial, which may be explained by the high activity of bendamustine in FL.^21,22^ Another single-arm prospective clinical trial of Pola-R-Benda in Japanese patients with relapsed/refractory DLBCL reported a median PFS of 5.2 months (95% CI: 3.6–not reached) at a median follow-up of 5.4 months.^23^

Although these data derive from DLBCL and FL but not MCL, the broad expression of CD79b provides biological plausibility for efficacy in MCL.^24^ The dosing regimen used in our patient corresponds to widely adopted standards (rituximab 375 mg/m^2^ day 1, bendamustine 90 mg/m^2^ days 2 and 3 of cycle 1, polatuzumab vedotin 1.8 mg/kg day 2 of cycle 1 and day 1 of subsequent cycles, 21-day cycles) and was well tolerated, with only transient grade 2 diarrhea and no treatment-related serious adverse events. Treatment resulted in rapid PET-negativity after three cycles and sustained CR, despite early discontinuation after five cycles. The durability of remission supports the potential of this combination even in highly refractory settings.

Beyond its role as salvage therapy after CAR-T failure, the profound and durable response observed in our patient also raises the question whether polatuzumab-containing regimens could have utility earlier in the treatment sequence, particularly as bridging therapy prior to CAR-T cell infusion. Disease control before CAR-T therapy has emerged as an important prognostic factor in aggressive B-cell lymphomas, and reduction of tumor burden prior to infusion may improve CAR-T expansion, reduce toxicity, and potentially enhance clinical outcomes.^25^ Given the rapid metabolic response and favorable tolerability observed with Pola-R-Benda in our patient, this regimen may represent an attractive bridging strategy in relapsed/refractory MCL patients awaiting CAR-T manufacturing or infusion. Importantly, however, bendamustine-containing regimens may impair T-cell fitness and have previously been associated with reduced CAR-T expansion and inferior outcomes.^26^ Therefore, the optimal timing, composition, and immunologic impact of polatuzumab-based bridging approaches in MCL remain uncertain and require prospective evaluation. Nevertheless, ADC-based bridging strategies may warrant further investigation, particularly in patients with high tumor burden or rapidly progressive disease requiring effective interim disease control.

Direct comparison of this case with published MCL is challenging due to paucity of evidence in the post-CAR-T failure setting. Nevertheless, the favorable long-term outcome observed stands in contrast to the typically dismal prognosis and suggests that a subset of MCL patients may derive substantial benefit from this regimen that may overcome profound refractoriness, as observed in DLBCL. However, extrapolation from DLBCL to MCL must be approached cautiously, given the distinct biology of MCL including the t(11;14) translocation, cyclin D1 overexpression, proliferation index variability, and disease heterogeneity.^4^

Mechanistically, several factors may have contributed to the therapeutic success in this patient. First, CD79b expression is preserved in most MCL, and antigen escape, a major mechanism of relapse after CAR-T therapy, is not commonly described for nontargeted antigens such as CD79b in our case. This preserved targetability may facilitate ADC activity even after CAR-T failure. Second, the quality of response and timing of relapse after CAR-T therapy is prognostically relevant. In the DESCAR-T registry, relapses within 3 months were associated with median OS of only 1.8 months, whereas relapses beyond 6 months conferred significantly better survival (median 9.0 months).^19^ Our patient achieved CR with Brexu-Cel and relapsed after 11 months, which may therefore reflect a less aggressive biological behavior and greater susceptibility to salvage treatment. Third, prior exposure to bendamustine is known to impair T-cell fitness and may blunt CAR-T expansion and efficacy, as described in ZUMA-2,^12^ but whether it alters sensitivity to bendamustine-containing regimens in the post-CAR-T setting is unknown. Finally, our patient received ibrutinib for almost 4 years and remained in CR during treatment. Ibrutinib was stopped due to patient compliance rather than MCL progression, and salvage treatment was initiated early after progression to CAR-T therapy and within a structured clinical protocol, which may have furthermore contributed to the patient’s favorable outcome. BTKi rechallenge after CAR-T relapse was considered in our patient, but due to previously noted incompliance on ibrutinib therapy, both physicians and the patient opted for treatment with Pola-R-Benda.

Despite the encouraging results, important considerations must be emphasized. This is a single-patient observation, and the sustained CR may reflect favorable underlying disease biology rather than generalizable efficacy. No biomarker currently exists to predict which post-CAR-T patients may benefit from Pola-R-Benda. Further, the advent of BTKi such as acalabrutinib, zanubrutinib, and pirtobrutinib is likely to substantially reshape the therapeutic landscape of both newly diagnosed and relapsed/refractory MCL in the near future. Acalabrutinib monotherapy has demonstrated meaningful activity in patients with relapsed/refractory MCL, with the phase II ACE-LY-004 trial reporting an overall response rate of 81% and a median progression-free survival of 22 months.^27^ It has also moved into the first-line setting combined with R-Benda in patients ⩾65 years based on the promising results of the phase III ECHO trial.^28^ Zanubrutinib, another highly selective BTKi, showed comparable efficacy as monotherapy in the BGB-3111-206 study, achieving an ORR of 84% and a CR rate of 69% in relapsed/refractory MCL.^29^ Most recently, the noncovalent BTKi pirtobrutinib demonstrated promising results in heavily pretreated MCL patients intolerant to prior covalent BTKi exposure, with an ORR of 57% and a median duration of response of 17.6 months in the phase I/II BRUIN trial.^30^ As these agents increasingly enter earlier lines of therapy, their use may significantly alter treatment algorithms and post-BTKi disease biology. Importantly, our patient was treated with ibrutinib as the sole BTKi and did not receive any next-generation BTKi, which may limit the generalizability of the current case in the context of evolving treatment standards. However, next-generation BTKis may be a promising next-line of therapy in our patient, in the event of relapse after Pola-R-Benda. Importantly, the role of consolidation therapies following ADC-based salvage regimens such as maintenance rituximab after Pola-R-Benda or immediately after ASCT remains undefined.

With regard to adverse events, the reported patient did not experience cytopenias, infections, or worsening peripheral neuropathy on treatment, which are commonly encountered with polatuzumab vedotin. Although the myocardial infarction in our patient was clinically most consistent with progression of advanced preexisting multivessel CAD, potential cardiotoxicity associated with ADCs warrants careful consideration. A recent retrospective pharmacovigilance and network meta-analysis evaluated cardiac adverse events across currently approved ADCs and demonstrated signals for cardiac adverse events for virtually all ADCs, including polatuzumab vedotin.^31^ However, reported cardiac events encompassed heterogeneous entities including heart failure, arrhythmias, or ischemic syndromes, and retrospective analyses are inherently limited in showing associations rather than causality. In the present case, the presence of severe, heavily calcified three-vessel CAD together with a substantial smoking history provides a strong alternative explanation for the cardiac event. Nevertheless, this case underlines the importance of cardiovascular risk stratification and close monitoring in patients receiving ADC-based regimens, particularly in individuals with established cardiovascular risk factors.

In conclusion, this case demonstrates that Pola-R-Benda can achieve durable remission after CAR-T failure in MCL, a setting of high unmet clinical need. These findings support further investigation of ADC-based regimens as salvage options after CAR-T failure. Until prospective data become available, Pola-R-Benda may be considered a rational individualized salvage strategy, particularly for younger and fit patients, but prospective evidence is required before definitive conclusions can be drawn. Our results furthermore encourage inclusion of post-CAR-T MCL patients in salvage trials of Pola-R-Benda or other novel strategies.

## Supplemental Material

sj-pdf-1-tam-10.1177_17588359261472319 – Supplemental material for Long-term complete remission following treatment with polatuzumab vedotin, rituximab, and bendamustine in a patient with mantle cell lymphoma relapsing after CAR-T cell therapy: a case report Supplemental material, sj-pdf-1-tam-10.1177_17588359261472319 for Long-term complete remission following treatment with polatuzumab vedotin, rituximab, and bendamustine in a patient with mantle cell lymphoma relapsing after CAR-T cell therapy: a case report by Vincent Sunder-Plassmann, Barbara Kiesewetter and Markus Raderer in Therapeutic Advances in Medical Oncology

## Declarations

**Ethics approval and consent to participate:** The present patient consented to participation within a clinical trial program (NCT05868395) on Pola-R-Benda in MCL. Via trial registration and patient enrollment, consent to publication and approval of the local ethics committee was obtained.

**Consent for publication:** By participating in the clinical trial program, consent for publication as individual case report and following manuscripts was obtained.

**Availability of data and materials:** Data may be made available upon reasonable request to the corresponding author, contingent upon approval by the institutional review board and completion of a data sharing agreement.*
