# Supplementary material for: Long-term complete remission following treatment with polatuzumab vedotin, rituximab, and bendamustine in a patient with mantle cell lymphoma relapsing after CAR-T cell therapy: a case report
Source: Ther Adv Med Oncol. 2026 Aug 5;18:17588359261472319. doi: 10.1177/17588359261472319 (PMC13443373; doi:10.1177/17588359261472319)
Supplement: Supplementary file 1 [file sj-pdf-1-tam-10.1177_17588359261472319.pdf]

CARE case report guidelines

CARE Checklist of information to include when writing a case report

(cc) BY-NC-ND

Topic	Item	Checklist item description	Reported on Line
Title	1	The diagnosis or intervention of primary focus followed by the words "case report"	1-3
Key Words	2	2 to 5 key words that identify diagnoses or interventions in this case report, including "case report"	16
Abstract (no references)	3a	Introduction: What is unique about this case and what does it add to the scientific literature?	18-22
	3b	Main symptoms and/or important clinical findings	22-28
	3c	The main diagnoses, therapeutic interventions, and outcomes	28-32
	3d	Conclusion—What is the main "take-away" lesson(s) from this case?	31-35
Introduction	4	One or two paragraphs summarizing why this case is unique (**may include references**)	61-66
Patient Information	5a	De-identified patient specific information.	69-70
	5b	Primary concerns and symptoms of the patient.	90-91
	5c	Medical, family, and psycho-social history including relevant genetic information	120-123, 125-127
	5d	Relevant past interventions with outcomes	70-89
Clinical Findings	6	Describe significant physical examination (PE) and important clinical findings.	69-70
Timeline	7	Historical and current information from this episode of care organized as a timeline	NA
Diagnostic Assessment	8a	Diagnostic testing (such as PE, laboratory testing, imaging, surveys).	89-99
	8b	Diagnostic challenges (such as access to testing, financial, or cultural)	NA
	8c	Diagnosis (including other diagnoses considered)	95
	8d	Prognosis (such as staging in oncology) where applicable	61-64
Therapeutic Intervention	9a	Types of therapeutic intervention (such as pharmacologic, surgical, preventive, self-care)	100
	9b	Administration of therapeutic intervention (such as dosage, strength, duration)	100-103
	9c	Changes in therapeutic intervention (with rationale)	107-132
Follow-up and Outcomes	10a	Clinician and patient-assessed outcomes (if available)	103-104
	10b	Important follow-up diagnostic and other test results	132-134
	10c	Intervention adherence and tolerability (How was this assessed?)	103-107, 132-135
	10d	Adverse and unanticipated events	104-131, 134-135
Discussion	11a	A scientific discussion of the strengths AND limitations associated with this case report	211-234
	11b	Discussion of the relevant medical literature **with references**.	137-191
	11c	The scientific rationale for any conclusions (including assessment of possible causes)	192-210
	11d	The primary "take-away" lessons of this case report (without references) in a one paragraph conclusion	250-257
Patient Perspective	12	The patient should share their perspective in one to two paragraphs on the treatment(s) they received	NA
Informed Consent	13	Did the patient give informed consent? Please provide if requested	Yes ☒ No ☐
